# A comparison of computer‐controlled versus manual on‐line patient setup adjustment

**DOI:** 10.1120/jacmp.v3i3.2571

**Published:** 2002-06-01

**Authors:** Kristy K. Brock, Daniel L. McShan, James M. Balter

**Affiliations:** ^1^ Department of Radiation Oncology University of Michigan Medical Center Ann Arbor Michigan 48109‐0010

**Keywords:** remote couch control, portal imaging, on‐line setup adjustment

## Abstract

A study was performed to determine the relative advantage of computer‐controlled couch movement versus manual repositioning to correct patient setup error measured using an electronic portal imaging device (EPID). Twenty‐eight on‐line setup adjustment trials of anterior‐posterior (AP) pelvic projections were evaluated, with 13 setups corrected by automated couch movement determined by direct feedback from the EPID image alignment tool and 15 setups manually corrected based on the transformation displayed from the same tool. The speed of setup adjustment and accuracy of corrected setup were determined. Computer controlled setup adjustment was determined to be faster (25.4 s versus 101.9 s) and slightly more accurate (1.8 mm versus 2.5 mm error in adjusted setup) than manual correction.

PACS number(s): 87.57.–s, 87.53.–j

## INTRODUCTION

Developments leading to improved image quality for electronic portal imaging devices (EPIDs) as well as image evaluation software may permit routine on‐line measurement and correction of patient setup. To date, limited trials of on‐line setup correction have been performed. Early studies of visual assessment of portal images followed by manual setup adjustment^1^ indicated that both the speed and final accuracy were prohibitive to routine on‐line use of this technology. Trials using image alignment software and a remote couch pendant^2^ ^,^ ^3^ showed improved setup accuracy and validated the benefits of quantitative image alignment tools, although treatment times were still dramatically increased using these tools. EPIDs currently suffer from a lack of integration with the control systems of linear accelerators (linacs). In addition, most commercially available linacs do not provide the ability to automatically move the treatment couch with precision. The purpose of this study is to determine whether removing these obstacles makes a significant impact on the speed and accuracy of on‐line setup adjustment.

## MATERIALS AND METHODS

A commercial video‐based EPID (Theraview, Infimed, Liverpool, NY) has been installed on one gantry of a racetrack microtron accelerator (MM50, Scanditronix, Uppsala, Sweden). The software control system has been replaced by an in‐house package that provides image acquisition and alignment tools.^4^ This software package communicates directly over an ethernet connection with an in‐house computer‐controlled radiotherapy system (CCRS),^5^ providing direct control of all treatment setup parameters, including treatment couch position. Using this system, transformations determined from alignment of reference and portal images can be automatically sent to the accelerator control computer. The CCRS combines the current table position with the planar alignment transformation (transformed into couch components based on the gantry angle of the portal projection), and determines a new setup table position. This position is set automatically the next time a therapist enters the treatment room and engages the enable bar on the accelerator control pendant.

A study was performed on treatment sessions which were proceeded by acquisition and analysis of an anterior‐posterior (AP) electronic portal image, and patient realignment if necessary. Such sessions included the first day of treatment and daily treatment on patients that presented problems in daily setup (e.g., obese patients). In this trial, two different modes of operation were compared: (1) automated adjustment of the treatment couch as described above, and (2) manual adjustment of the couch based on the alignment transformation determined from the EPID. Automated setup adjustment was performed 13 times on 13 different patients. Manual setup adjustment was performed 15 times on 14 patients (one patient underwent manually setup adjusted on two different days).

## ANALYSIS OF SPEED

The CCRS maintains a record of all events associated with accelerator operation, with associated digital timestamps. This record was used to reconstruct the relative amount of time required for each step in the process of on‐line setup evaluation and adjustment. The process for automated setup adjustment was marked by the following times:

$A_{\text{image}}$‐The time of image acquisition (initiation of BEAM__ON)

$A_{\text{ISO}}$‐The time at which the transformation was received by the CCRS

$A_{\text{next}}$‐The time at which the next BEAM__ON event occurred

The only activity that occurred between $A_{\text{image}}$ and $A_{\text{ISO}}$ is the alignment of the acquired image to the reference image. Therefore the time required for image alignment can be calculated by

(1) $$T_{\text{align}}=A_{\text{ISO}}-A_{\text{image}}.$$

For the process of manual setup adjustment, the times had a slightly different representation. The manual setup adjustment process included a step in which the therapist, after entering the treatment room and adjusting couch position, saved the new reference couch setup by pressing a button sequence on the control pendant. Under this process the times recorded were:

$M_{\text{image}}$‐The time of image acquisition (initiation of BEAM–ON)

$M_{\text{ISO}}$‐The time at which the new reference couch position was recorded by the CCRS

$M_{\text{next}}$‐The time at which the next BEAM–ON event occurred

The time required for the sequence of events following couch adjustment up to the delivery of the next radiation event is defined as

(2) $$T_{\text{sequence}}=M_{\text{next}}-M_{\text{ISO}}.$$

$A_{\text{ISO}}$ occurs before the therapist enters the treatment room and moves the couch. $M_{\text{ISO}}$ occurs after the therapist enters the treatment room. The time from $A_{\text{ISO}}$ to $A_{\text{next}}$ reflects the therapist entering the treatment room and enabling couch movement, leaving the room, and the control sequence for enabling the next BEAM_ON event. The time from $M_{\text{ISO}}$ to $M_{\text{next}}$ reflects the therapist leaving the room and the control sequence for the next BEAM ON event. The image alignment time and the–steps following couch adjustment are expected to be the same for manual and automated adjustment. The average value of $T_{\text{align}}$ can be calculated from the automated cases and the average value of $T_{\text{sequence}}$ can be calculated from the manual cases. These values can then be used to calculate the amount of time spent adjusting the patient for each of the automated and manual cases. Automated adjustment time can be calculated via

(3) $$A_{\text{adjust}}=(A_{\text{next}}-A_{\text{ISO}})-[T_{\text{sequence}}].$$

Manual adjustment time can be calculated via

(4) $$M_{\text{adjust}}=(M_{\text{ISO}}-M_{\text{image}})-[T_{\text{align}}].$$

These times were calculated from CCRS records of treatments. To estimate the validity of the calculation of [$T_{\text{align}}$] and [$T_{\text{sequence}}$], times were recorded for patients that were imaged and determined following alignment to not require setup adjustment (a threshold was used to determine whether the setup error was to be corrected.). The nonadjusted patients had times recorded at $A_{\text{next}}$ and $A_{\text{image}}$ or ($M_{\text{next}}$ and $M_{\text{image}}$). In these cases, there is no $A_{\text{ISO}}$ or $M_{\text{ISO}}$ time. If our calculations for $T_{\text{sequence}}$ and $T_{\text{align}}$ are valid, their sum should be equivalent to the time spanned between $A_{\text{image}}$ and $A_{\text{next}}$ or ($M_{\text{image}}$ and $M_{\text{next}}$), as the only tasks performed during this time are the sequence and the alignment.

(5) $$T_{\text{sequence}}+T_{\text{align}}=A_{\text{next}}-A_{\text{image}}(\text{or}\;\;M_{\text{next}}-M_{\text{image}})$$

### Analysis of accuracy

Accuracy of setup adjustment was determined from offline analysis of portal images taken prior to adjustment as well as verification images or films acquired following setup adjustment for patients. If films were used, they were digitized and imported into the analysis software. Two separate observers analyzed each image. To reduce the effect of random error in image alignment on the accuracy estimates, both observers aligned each image twice. The resulting average of all four retrospective alignment measurements was taken as the residual setup error of the patient. Assuming that the error in measurement is randomly distributed, such an average may reduce the impact of measurement error by a factor of 2.

The control system for the treatment couch on the MM50 generates movements in increments of 1 mm. Thus, a correction in setup error based on a finer measurement is quantized to the nearest 1 mm. It can be seen that there are four values determined for automated setup adjustment:

$A_{\text{initial}}$ is the initial patient position (evaluated offline as described above) prior to adjustment.

$A_{\text{action}}$ is the actual implemented adjustment (quantized in 1 mm steps).

$A_{\text{final}}$ is the final patient position (evaluated offline as described above).

Based on these values, the error in correcting patient position can be stated as

(6) $$A_{\text{error}}=A_{\text{final}}-(A_{\text{initial}}-A_{\text{action}}).$$

$A_{\text{error}}$ may have components due to measurement error, both in retrospective alignment of the final position and alignment at the time of on‐line analysis, as well as couch correction. Patient motion between image acquisitions could also cause error in final positioning.

For practical manual setup correction, the adjustments that the technologists made were quantized to values of 3,5,8,10,15,… mm. The initial alignment values were typically rounded to the nearest quantization value. Values for manual adjustment accuracy can thus be defined as:

$M_{\text{initial}}$ is the initial patient position (evaluated offline as described above) prior to adjustment.

$M_{\text{action}}$ is the actual implemented adjustment (instruction given to the technologist).

$M_{\text{final}}$ is the final patient position (evaluated offline as described above).

And the error in correcting patient position manually is

(7) $$M_{\text{error}}=M_{\text{final}}-(M_{\text{initial}}-M_{\text{action}}).$$

## RESULTS

Twenty‐eight adjustments were made. Of these, 13 adjustments were made using the automated couch adjustment and 15 were made using manual correction. The speed and accuracy of the two methods were compared.

## RESULTS OF SPEED ANALYSIS

[$T_{\text{align}}$] was determined from an average over 13 automated cases. The average time between the time of image acquisition (initiation of BEAM__ON) and the time at which the transformation was received by the CCRS, [$T_{\text{align}}$], was 156 sec (σ=59sec). [$T_{\text{sequence}}$] was determined from an average over 14 manual cases (time data from one manual case was not obtained). The average time between the time at which the new reference couch position was recorded by the CCRS and the time at which the next BEAM__ON event occurred, [$T_{\text{sequence}}$], was 167 sec (σ=88sec) (Table I).

**Table I:** Alignment time and sequence time.

	Average (s)	σ (sec)
$T_{\text{align}}$	156	59
$T_{\text{sequence}}$	167	88

The validity of the values of [$T_{\text{align}}$] and [$T_{\text{sequence}}$] were determined by Eq. (5). $A_{\text{next}}$–$A_{\text{image}}$ (or $M_{\text{next}}$–$M_{\text{image}}$) was calculated for eight cases. The average for these eight cases was 294 sec. The averages of $\left[T_{\text{align}}]+[T_{\text{sequence}}\right]$ was 323 sec (Table II). These values are not significantly different, suggesting that the calculated values of [$T_{\text{align}}$] and [$T_{\text{sequence}}$] are appropriate representations of actual process times.

**Table II:** Validity of [$T_{\text{align}}$ and [$T_{\text{sequence}}$].

	Average (sec)
$A_{\text{next}}-A_{\text{image}}$ (nonadjusted patients)	294
$\left[T_{\text{align}}]+[T_{\text{sequence}}\right]$	323

$A_{\text{adjust}}$ was then calculated from Eq. (3) for each of the 13 automated cases. The average $A_{\text{adjust}}$ was 25.4 sec (σ=48.6sec). Four of the calculated $A_{\text{adjust}}$ values were negative. This is due to the population sampling which determined the value of [$T_{\text{sequence}}$], from which $A_{\text{adjust}}$ was calculated. Therefore, in some individual instances, the $T_{\text{sequence}}$ for that case was less than the average, resulting in a negative alignment time. One factor contributing to these negative times is when an additional person aided in patient treatment, preparing the next event while the therapists adjusted the patient. This happened rarely and is not believed to be solely responsible for the negative adjustment times (Table III).

**Table III:** Automated adjustment times.

Case	$A_{\text{adjust}}$ (sec)
1	63
2	–15
3	11
4	18
5	106
6	84
7	–2
8	50
9	13
10	–79
11	–3
12	68
13	16
Average	25.4
St Dev	48.6

$M_{\text{adjust}}$ was then calculated from Eq. (4) for each of the 14 manual cases. One of the calculated $M_{\text{adjust}}$ values was negative. The average $M_{\text{adjust}}$ was 101.9 sec (σ=53.6sec) (Table IV).

**Table IV:** Manual Adjustment times.

Case	$M_{\text{adjust}}$ (sec)
1	66
2	70
3	90
4	142
5	89
6	101
7	61
8	214
9	180
10	90
11	135
12	81
13	111
14	–3
Average	101.9
St Dev	53.6

The difference between the average $A_{\text{adjust}}$ and $M_{\text{adjust}}$ was 76.5 sec.

## RESULTS OF ACCURACY ANALYSIS

Accuracy in patient correction was determined for 13 automated cases and 15 manual cases. The patient correction error for automated setup adjustment was 1.84 mm (σ=0.51 mm; range =1.00 to2.43 mm) (Table V). The patient correction error for manual setup adjustment was 2.49 mm (σ=1.30 mm; range=1.20 to5.41 mm). The largest vector correction made to a patient was 34.5 mm.

**Table V:** Automated and manual accuracy.

Averages	Automated (mm)	Manual (mm)
Patient correction error (σ)	1.84 (0.51)	2.49 (1.30)
Average residual error (σ)	2.56 (0.71)	3.10 (1.30)
Average residual error‐corrected for quantization (σ)	2.56 (0.71)	2.21 (1.47)

The average residual error is the offset of the patient from their desired position at the time of treatment. The average residual error does not correct for the inaccuracy in the initial alignment of the patient or the quantization of the transformation given to the therapist in the manual adjustment. The average residual error for automated and manual cases were 2.56 mm (σ=0.71 mm) and 3.10 mm (σ=1.30 mm), respectively. To correct for the quantization of the transformation given to the therapist during manual adjustment, the difference between the initial alignment and the instructed move was subtracted from the residual error of the final patient position. Correcting for the quantization results in an average residual error–corrected for quantization of 2.21 mm (σ=1.47 mm) (Table V).

## DISCUSSION

The benefits of implementation of daily imaging and setup adjustment are contrasted by the time required to perform the necessary imaging and adjustment. Historically, advancements in accuracy come at the price of longer time requirements. This study investigated the improvements in accuracy and reduction in time using computer controlled setup adjustment as an aid to online image‐guided positioning. Setup errors were determined only in 2D and only in the left‐right and inferior‐superior directions, as only anterior‐posterior images were acquired. Neither in‐plane nor out‐of‐plane rotations were analyzed. Within these limits, computer‐controlled setup adjustment is shown to be faster and slightly more accurate than manual setup adjustment. Automated repositioning also overcame a limit of the accelerator control system that necessitated extra time for repositioning. During the automated process, while one technologist is in the treatment room enabling the setup adjustment, another could begin the beam sequence to prepare for treatment.

This is not an option in manual setup adjustment because the treatment machine must be in standby mode during the repositioning, making it impossible to begin the beam sequence. We expect these findings to be similar for 3D setup adjustment.

Similar studies have also been performed to evaluate the time necessary to perform on‐line patient imaging and automatic table adjustment. Van de Steene *et al*.^3^ used on‐line localization with an EPID and a remote couch control and found the mean time for both the measurement and correction procedure to be 2.5 min. After correction less than 10% of the images had a residual error greater than the threshold for adjustment. Less than 25% of the cases when the original setup error did not exceed the threshold were later determined to have a residual error exceeding the threshold. The time required for measurement and adjustment was slightly faster, 30 sec, than our system.

De Neve *et al*. performed two studies.^6^ ^,^ ^7^ The first utilized a fluoroscopic on‐line portal imaging system and remote couch adjustment. Patients involved in this study included head and neck, thorax, and pelvis. The fraction of total treatment time for comparison and adjustment of all patients had a median of 30.7%. For pelvic patients alone, the median fraction of total treatment time was 29.8%. The second used the same fluoroscopic on‐line imaging system and remote couch adjustment on 13 pelvic patients. The treatment time was increased by a median of 36.5% and over 80% of patient setups were corrected. However, both of these studies used visual comparison between the portal image and a simulator film and although the couch adjustment was made from outside the room, it was still a manual adjustment. The fraction of total treatment time for measurement and adjustment in this study was similar to ours. Their study took 9.7% more treatment time than our study, 29.8% compared to 20.1%.

Van den Heuvel *et al*.^2^ investigated the clinical implementation of intra‐treatment correction using an EPID under an objective computer‐aided protocol. The time added to treatment due to interaction with the EPID was a mean of 57.6%. The image analysis procedure added approximately 11.1% to the treatment time and adjusting the patient with the remote couch controller added 36.6%. A threshold for adjustment was set at 2 mm and the residual errors had a standard deviation of less than 2 mm. The time added to treatment for interaction with the EPID, image analysis, and couch adjustment was slightly different than our study, 20.2%, 17.3%, and 2.8%, respectively (of a baseline average treatment time of 900 seconds). The residual errors also had similar standard deviations.

Herman *et al*.^8^ used a computer controlled fluorescent screen‐mirror imaging system with physician's visual comparison to evaluate patient offset. Patient offset greater than 5 mm were corrected. 18% of the final images had errors exceeding the threshold for corrections.

Gilhuijs *et al*. performed two studies.^9^ ^,^ ^10^ The first involved chamfer matching to determine errors in translation, rotation, and magnification. An average accuracy 1.8 mm was found for the pelvic region. The computational time required for the analysis took an average of 3 s. The second study used digital portal images and CT data to determine prostate and parotid glad patient setup in 3D. Using fast computation of digitally reconstructed radiographs an accuracy of the order of 1 mm and 1° (SD) could be obtained. The complete measurement process required approximately 10 min. This study included 3D analysis, where our study was limited to 2D, although the accuracy was comparable between the two.

Recently Bel *et al*.^11^ investigated the clinical feasibility of a computerized remote control for a Siemens ZXT treatment couch. The average precision of the reading of the relative table translation was 0.25 mm (SD). The accuracy of shifts in one direction was 0.6 mm (SD) in each direction and 0.04° (SD) for rotations. The accuracy of the 3D shifts in 3D vector length was 0.96 mm (SD). 95% of 3D adjustments with rotations were completed in less than 16 s. The average time required for 2D automated adjustment using our system was 6 s longer, with an average of 22.4 sec.

Since this study was performed, the computer‐controlled setup adjustment method has been a vital part of other studies in the clinic. A “tilt and roll” device has been added to perform automated 3D daily target volume positioning.^12^ ^–^ ^14^ Trials are ongoing for online 3D adjustment in the prostate and liver.

## ACKNOWLEDGMENTS

This work was supported by a grant from the Whitaker Foundation. Dr. Balter was also supported as a Kimmel Scholar.
